# Norvaline Restores the BBB Integrity in a Mouse Model of Alzheimer’s Disease

**DOI:** 10.3390/ijms20184616

**Published:** 2019-09-18

**Authors:** Baruh Polis, Vyacheslav Gurevich, Michael Assa, Abraham O. Samson

**Affiliations:** 1 Drug Discovery Laboratory, The Azrieli Faculty of Medicine, Bar-Ilan University, Safed 1311502, Israel; avraham.samson@biu.ac.il; 2 Laboratory of Cancer Personalized Medicine and Diagnostic Genomics, The Azrieli Faculty of Medicine, Bar-Ilan University, Safed 1311502, Israel; slavagur13@gmail.com; 3 Inter-laboratory Equipment Center, The Azrieli Faculty of Medicine, Bar-Ilan University, Safed 1311502, Israel; michael.assa@biu.ac.il

**Keywords:** Alzheimer’s disease, angiopathy, BBB, norvaline, arginase, arginine, NOS, NO

## Abstract

Alzheimer’s disease (AD) is a chronic neurodegenerative disorder and the leading cause of dementia. The disease progression is associated with the build-up of amyloid plaques and neurofibrillary tangles in the brain. However, besides the well-defined lesions, the AD-related pathology includes neuroinflammation, compromised energy metabolism, and chronic oxidative stress. Likewise, the blood–brain barrier (BBB) dysfunction is suggested to be a cause and AD consequence. Accordingly, therapeutic targeting of the compromised BBB is a promising disease-modifying approach. We utilized a homozygous triple-transgenic mouse model of AD (3×Tg-AD) to assess the effects of L-norvaline on BBB integrity. We scrutinized the perivascular astrocytes and macrophages by measuring the immunopositive profiles in relation to the presence of β-amyloid and compare the results with those found in wild-type animals. Typically, 3×Tg-AD mice display astroglia cytoskeletal atrophy, associated with the deposition of β-amyloid in the endothelia, and declining nitric oxide synthase (NOS) levels. L-norvaline escalated NOS levels, then reduced rates of BBB permeability, amyloid angiopathy, microgliosis, and astrodegeneration, which suggests AD treatment agent efficacy. Moreover, results undergird the roles of astrodegeneration and microgliosis in AD-associated BBB dysfunction and progressive cognitive impairment. L-norvaline self-evidently interferes with AD pathogenesis and presents a potent remedy for angiopathies and neurodegenerative disorders intervention.

## 1. Introduction

Alzheimer’s disease (AD) is an incurable chronic neurodegenerative disorder and the most common type of dementia [1]. The disease development is characterized by progressive brain atrophy, amyloid-beta (Aβ) deposition, and neurofibrillary tangles (NFT) formation throughout the brain parenchyma [2]. Despite a century-long investigation and recent significant evolution in our understanding of the disease pathogenesis, there is no complete scientific consensus concerning the causes of the disease, which seriously hinders the search for AD-modifying remedies.

Besides the brain parenchyma, the Aβ peptides deposit extensively in the vessel walls, which causes cerebral amyloid angiopathy (CAA) [3]. A remarkable experiment applying in vivo imaging of CAA in seven-month-old AD mice by Kim et al. (2015) demonstrated the Aβ deposits wrapped around the vessel wall in patches [4]. Of note, plaques do not form complete rings at this stage and are not detectable in either the dura vessels or veins. Other studies have shown prominent cerebral amyloid angiopathy in transgenic mice overexpressing human APP in neurons [5].

It is noteworthy that AD patients exhibit diverse central nervous system (CNS) vascular pathologies. Commonly, their central cerebral arteries contain atherosclerotic plaques, intracerebral penetrating arteries have substantially thinner muscle layers and contain amyloid plaques, while small arterioles and capillaries display endothelial degeneration and are surrounded by amyloid deposits [6]. Advanced intracranial atherosclerosis leads to chronic brain hypoperfusion, oxidative stress, and, eventually, dementia [7]. Additionally, AD-related angiopathy is associated with a meaningfully increased blood–brain barrier (BBB) permeability, which correlates with the severity of cognitive decline and cerebrospinal fluid (CSF) albumin concentrations [8]. Accordingly, BBB dysfunction has been proposed as a leading cause and a characteristic consequence of AD-related pathology [9,10].

Recent longitudinal studies followed by postmortem brain examinations have proven a strong association of CAA pathology with aging and AD [11]. Consequently, age-dependent BBB leakage was shown in various rodent models of AD, and BBB permeability index was suggested to characterize CAA progression and serve as a surrogate marker for treatment response [12]. Of note, CAA is an independent risk factor of cognitive dysfunction [13]. However, angiopathy contributes to the clinical presentation of dementia by interacting with other CNS pathologies and further aggravates the cognitive impairment [14].

The BBB is a specific term, which describes the distinctive properties of the CNS microvasculature. The chief function of the BBB is to regulate the precise movement of cells and molecules between the CNS and the blood in accordance with the functional needs. In view of that, it is an extremely specialized and highly selective semipermeable border separating the blood from the brain and extracellular fluid [15]. 

Endothelial cells (ECs) form the inner walls of the brain’s blood vessels and serve as the primary BBB anatomic unit [16]. Astrocytic glial cells extend cellular processes to ensheath the vascular tube and provide a cellular connection between the neuronal circuitry and blood vessels [15]. This particular morphological feature assists in the regulation of the blood flow to meet the requirements of constantly changing neuronal activity [17]. Neurons, astrocytes, ECs, myocytes, pericytes, and extracellular matrix components compose the neurovascular unit (NVU), which is a functional element identifying the needs of neuronal supply and triggering the necessary responses for such demands [18]. Astrocytic cells composing the NVU continuously exchange metabolic substrates with cerebral microvessels and, in parallel, release potent vasodilators like prostaglandin E2 and epoxyeicosatrienoic acids, as well as vasoconstrictors like arachidonic acid, following the metabolic requirements [19]. Therefore, the mammalian brain is capable of radically increasing the blood flow, glucose, and oxygen uptake in its functionally active regions [18].

Astrocytes secrete substantial quantities of Aβ and contribute to AD-associated overall amyloid burden. Moreover, reactive astrocytes meaningfully escalate their production rate of APP and β-site APP cleaving enzyme 1 (BACE1) [20], which further aggravates the Aβ-related pathology. Astrocytes additionally play a central role in the brain Aβ clearance via modulating several Aβ-degrading enzymes and critical cellular degradation pathways [21]. Therefore, the normal functioning of these cells is critical for both BBB integrity and Aβ clearance. Consequently, severe endothelial and astrocytic dysfunctions play a causative role in AD-related energy metabolism disturbances and chronic oxidative stress, which, together with other factors, contribute to AD pathogenesis and, eventually, clinical dementia development.

It is worth highlighting that AD is characterized histopathologically by both astrogliosis and astrodegeneration, depending on the stage of the disease and the brain region [22]. In animal models, these pathologies have been shown to precede the well-known hallmarks, including amyloid plaques and NFT [23]. Moreover, in the cortex and hippocampus of the murine models of AD, as well as in human AD brains, CAA is followed by a considerable decline in the numbers of astrocytic processes contacting the vasculature [24]. Therefore, AD-associated astrocytic atrophy and degeneration leads to reduced coverage of blood vessels and synapses, and eventually, to a chronic NVU dysfunction and BBB breakdown [19].

Remarkably, typical astroglial degeneration, with a substantial reduction in the number of extending processes and independent of Aβ deposition, is prominent in the entorhinal cortex of one-month-old and the prefrontal cortex of three-month-old homozygous triple-transgenic mice models of Alzheimer’s disease (3×Tg-AD) [25]. Astrodegeneration is prominent in the hippocampus of the twelve-month-old animals [26]. Our previously published results demonstrate that hippocampi (CA4 area) of seven-month-old 3×Tg-AD mice are characterized by severe astrodegeneration, even without significant changes in the astrocytes density [27].

In the present study, we used the same animal model and scrutinized the astrocytic cells in the vicinity of the vasculature, and report drastic alterations in astroglial morphology, which are apparent in the hippocampi of the 3×Tg-AD mice, compared to the wild-type (WT) animals. We utilized an immunohistochemical approach to determine the glial cytoskeleton domain and quantify the glial fibrillary acidic protein (GFAP) positive surface area, intensities, number of processes, and the spatial relationships between astrocytes and ECs in the NVU. Subsequently, we evidenced a significant effect of L-norvaline upon the GFAP-positive surface area, and intensity, in the NVU area. 

AD is characterized by the involvement of macrophage recruitment and microglial activation [28]. Blood-borne activated monocytes/macrophages transmigrate via disrupted BBB, phagocytize, and shuttle Aβ from neurons to vessels [29]. In general, stimulation of perivascular macrophage turnover results in better clearance of Aβ deposits [30]. However, in progressive AD, macrophages are deficient of Aβ clearance, due to reduced phagocytic function, and undergo apoptosis, which leads to a massive Aβ release into the vessel wall and, eventually, CAA exacerbation [29]. Moreover, perivascular macrophages are an immense source of reactive oxygen species mediating the neurovascular dysfunction and contributing to AD pathogenesis [31]. Accordingly, manipulating their function is a promising AD therapeutic strategy. 

**Figure 1:**
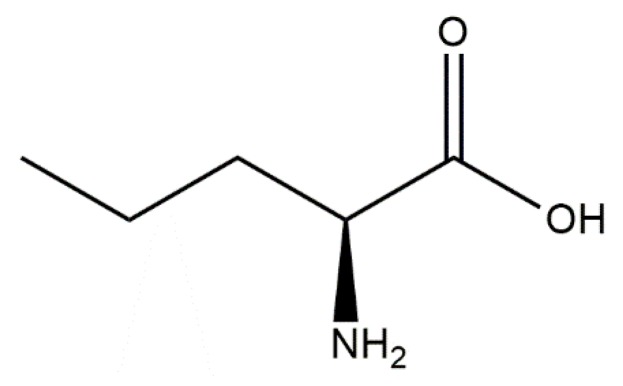
Chemical structure of L-norvaline.

L-norvaline (or 2-aminopentanoic acid) is a nonproteinogenic amino acid and an isoform of the common amino acid valine, which has been intensively investigated in early enzymological studies [32]. Structural similarity with L-ornithine (Figure 1) provides the substance with a competency of negative feedback arginase inhibition [33]. Additionally, the anti-inflammatory properties of L-norvaline via inhibition of ribosomal protein S6 kinase beta-1 have been demonstrated in vitro [34]. Of note, arginase inhibition has been proposed to reduce the risk and frequency of cardiovascular diseases [35]. Consequently, various arginase inhibitors have been investigated in rodent models and in humans, and L-norvaline—a noncompetitive arginase inhibitor—has attracted clinical interest.

Here, we demonstrate a substantial reduction in the rate of CAA, which is reflected by a significant decline in Aβ positivity following the treatment with L-norvaline. Additionally, we analyze CAA-associated and treatment-related alterations in the brain-resident innate immune cells. Finally, we propose a rational model, which explains the treatment-associated changes and paves new avenues in the AD research.

## 2. Results

### 2.1. Norvaline Improves the BBB Integrity in the 3×Tg-AD Mice

Recent in vivo studies in patients with early AD proved severe BBB failure, principally in the hippocampus [36], yet apparent in other brain regions as well [37], even prior to brain atrophy symptoms and clinical dementia manifestation. Animal models of AD develop an age-dependent BBB breakdown, which manifests by loss of tight junctions, basement membrane degeneration, perivascular immunoglobulin G accumulation, thrombin and fibrinogen deposition, leakage of Evans blue and other tracers [38].

**Figure 2:**
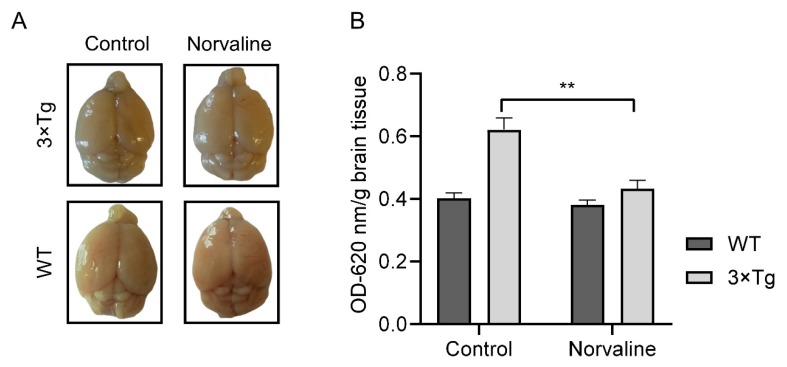
Norvaline reduces the blood–brain barrier (BBB) permeability. BBB integrity was evaluated via Evans blue BBB permeability assay. Homozygous triple-transgenic mice models of Alzheimer’s disease (3×Tg-AD) and wild-type (WT) mice were intraperitoneally injected with Evans blue dye (50 µg/g of body weight). Three hours post-injection, mice were deeply anesthetized, perfused with saline, and dye presence in the brains was measured at OD_620_. (**A**) Representative images of the control and norvaline-treated brains. (**B**) The brain homogenates of control and norvaline-treated mice were analyzed spectrophotometrically. The OD values at 620 nm were normalized by division by the weights of the appropriate brains and presented as means ± SEM, ** *p* < 0.01, *n* = 6.

Tibbling et al. (1977) pointed out that CNS albumin is derived from peripheral sources. Accordingly, the albumin index can indicate the rate of BBB disruption and facilitate the efficacy of routine clinical work [39]. Subsequently, several groups demonstrated elevated levels of CSF albumin in patients with AD [40,41]. We applied a traditional Evans blue assay to assess the albumin presence in the brain tissue. The mean measured supernatant optical density (OD_620_) per brain weight from WT control animals was 0.4 ± 0.02 (Figure 2B), which accords with the results from other groups that utilized the same protocol [42]. Remarkably, the mean OD_620_ of the supernatant from seven-month-old 3×Tg-AD controls increased up to 0.62 ± 0.04 and still was substantially lower than OD_620_ = 0.9 obtained from 15-month-old 3×Tg-AD mice [42], which points to gradual progressive severe age-dependent BBB defects in this particular AD model. Remarkably, L-norvaline treatment significantly (*p* < 0.01) improved the BBB integrity in the transgenic mice, as reflected by a reduction (by 31%) in the mean OD_620_ (0.43 ± 0.066). Of note, we did not observe any significant effect of the treatment upon the BBB permeability in the WT animals (Figure 2). The two-way ANOVA test was used to reveal a significant (F_(1,20)_ = 10.47 *p* = 0.0041) interaction (accounting for 14.21% of the total variance) between the genotype and the type of treatment, and their mutual effect upon the BBB permeability, which further indicates L-norvaline efficacy.

### 2.2. Norvaline Attenuates Cerebral Amyloid Angiopathy

#### 2.2.1. Norvaline Reduces Endothelial Amyloidosis

APP is an evolutionarily conserved protein with high expression levels in ECs of cerebral and peripheral arteries [43]. Recent data show that brain microvascular ECs express equivalent or rather higher levels of APP compared to the primary neurons [44]. Moreover, the same study proved that amyloidogenic BACE1 pathway is present in ECs and the ratio of endothelial Aβ42/Aβ40 is similar to that in neurons. Consequently, endothelial Aβ peptides deposit extensively in cerebral vessel walls.

**Figure 3:**
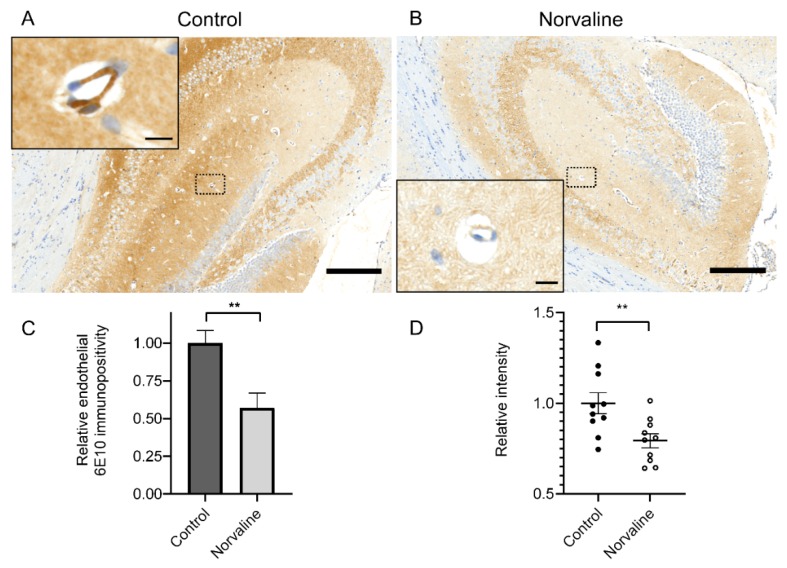
Cerebral amyloid angiopathy (CAA) in 3×Tg-AD mice revealed by 6E10 staining. (**A**) Representative × 40 photomicrograph demonstrating extensive 6E10 immunopositivity in the hippocampus of vehicle-treated seven-month-old mice (scale bar = 200 µm). (**B**) Representative × 40 photomicrograph with significantly reduced 6E10 immunopositivity in the hippocampus of L-norvaline treated mice (scale bar = 200 µm). Insets show hippocampal penetrating microvessels with the illustrative morphology at higher magnification (scale bar = 10 µm). (**C**) Quantification of the relative Aβ burden with ZEN 2.5 (6E10 immunopositive surface area) in the microvessels of control and norvaline-treated 3×Tg-AD mice. (**D**) Quantification of the relative stain intensity with ImageJ (n = 10, five mice for each group, two sections per mouse, one vessel per section, mean ± SEM, Student’s *t*-test, ** *p* < 0.01).

We applied an immunohistochemistry assay to analyze the effect of L-norvaline upon the rate of Aβ deposition in the 3×Tg-AD mice brain microvessels’ walls. Previously, we have shown a severe Aβ positivity decline in the cortices [27] and hippocampi [45] following the L-norvaline treatment. Here, we evidenced considerable levels of APP/Aβ in the cerebral microvessels’ ECs (Figure 3A), and demonstrated a substantial reduction in the levels of Aβ positivity following the treatment (Figure 3B).

The 6E10+ surface area in µm^2^ above a preset threshold (Figure 3C) has been divided by the vessel’s surface area in µm^2^. Therefore, we show a relative number, which better reflects the rate of adjusted immunopositivity. The image densitometry analyses confirm a significant effect of the treatment upon the rate of 6E10 endothelial immunopositivity and stain intensity (Figure 3C,D).

#### 2.2.2. Norvaline Diminishes the Rate of Astrodegeneration in the 3×Tg-AD Mice

**Figure 4:**
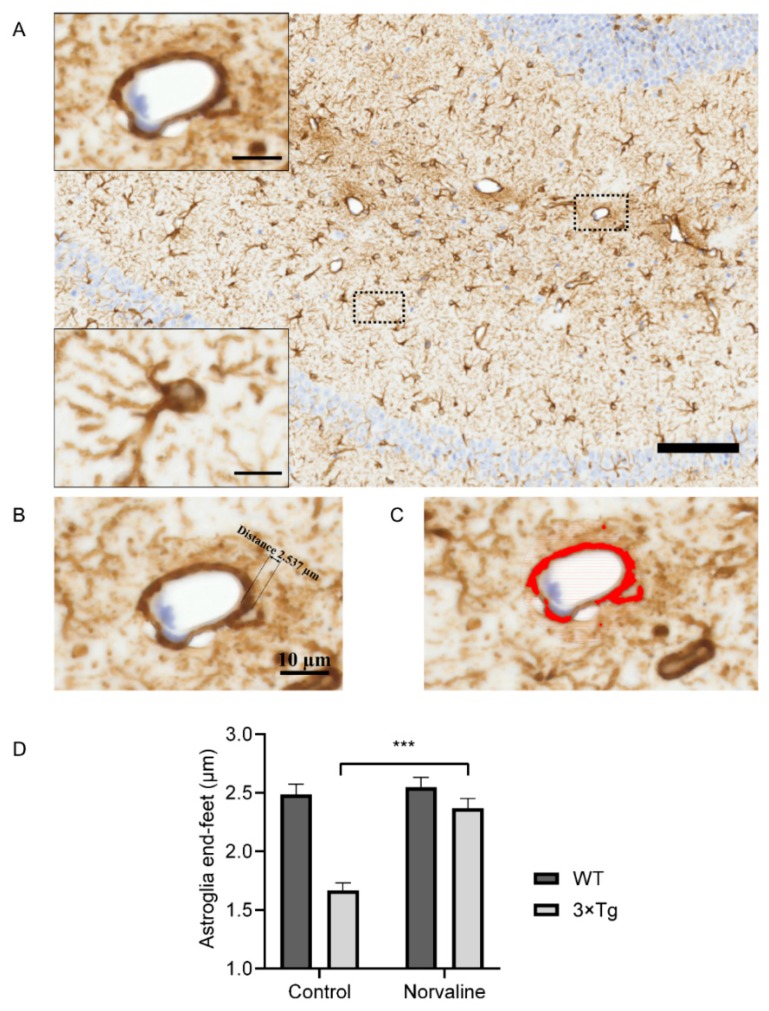
Representative hippocampal bright-field × 40 micrograph from WT control animal (scale bar = 100 µm) with insets showing a penetrating microvessel and a typical astrocytic cell in CA1 area with the illustrative morphology at ×100 magnification (scale bar = 10 µm) (**A**)**.** Measurement of the end-foot thickness (**B**) and glial fibrillary acidic protein (GFAP) immunopositivity (**C**) of neurovascular unit (NVU) within a typical microvessel by ZEN 2.5. (**D**) Quantitative analysis of the astroglia end-feet thickness in relation to genotype and type of treatment. Two-way ANOVA test graphical presentation, demonstrating a significant effect of the treatment upon astroglia NVU end-feet in 3×Tg-AD but not WT mice (*n* = 10 (5 mice for each group, two sections per mouse, 1 NVU per section)). Mean ± SEM, *** *p* < 0.001.

GFAP+ astrocytes are the most abundant brain cells (Figure 4A). They possess an extensive range of functions, including maintenance of the extracellular homeostasis and the BBB, together with metabolic support to neurons [46]. Astrocytic cells are connected to the neurovasculature via projections terminating in the end-foot (Figure 4B) and are tightly abutted and adherent to the basement membrane [47]. 

AD development is followed by complex changes in astrocytes morphology [20,48]. Of note, 3×Tg-AD mice generally display dystrophic astroglial phenotypes, which are associated with decreased levels of GFAP protein, GFAP immunoreactive surface area, and reduced number of astrocyte processes [25].

Here, we analyze the GFAP immunopositive surface area and optical density (Figure 4C) in the vicinity of hippocampal penetrating blood microvessels (Figure 4A) (about 10 µm in diameter). The relative thickness of the astroglia end-feet (Figure 4B) displayed significant genotype and treatment-related differences (Figure 4D). The two-way ANOVA test revealed a significant (F_(1,36)_ = 15.7 *p* = 0.0003) interaction (accounting for 13.94% of the total variance) between the genotype and the type of treatment, and their mutual effect upon the astroglia end-feet thickness. Remarkably, L-norvaline did not affect this parameter in WT mice but demonstrated a significant (*p* < 0.001) influence upon 3×Tg-AD animals with an increase (by about 42%) (Figure 4D).

**Figure 5:**
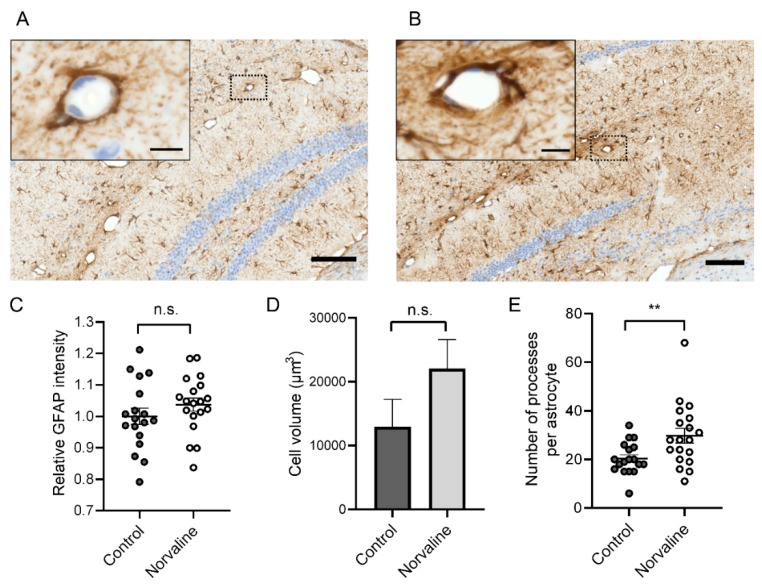
Representative hippocampal bright-field × 40 micrographs from 3×Tg-AD control (**A**) and L-norvaline treated (**B**) mice brain (scale bar = 100 µm) with insets showing penetrating microvessels at × 100 magnification (scale bar = 10 µm). Quantification of GFAP+ stain intensity (**C**), astrocyte cell volume (**D**), and the number of processes per astrocyte (**E**), examined with Imaris® and analyzed via Student’s unpaired *t*-test (*n* = 18 for control and *n* = 19 for treated mice), ns: not significant, ** *p* < 0.01.

Imaris® software has been applied for large-scale analysis of 3D astrocyte morphology. We reconstructed individual cells in the CA1 area (Figure 4A inset) and revealed a significant (by about 50%) increase (from 20.39 ± 1.55 to 29.79 ± 3.01) in the number of astrocytic protoplasmic processes following the treatment (Figure 5E). The analysis of the staining intensity (Figure 5C) and cell volume (Figure 5D) also demonstrated an increase in the L-norvaline treated group; however, the effect did not reach statistical significance. 

#### 2.2.3. Norvaline Moderates the Activity of the Brain Perivascular Macrophages

In the AD brains, macrophages have been shown to infiltrate extensively the perivascular space and neuropil. In particular, they are apparent at the sites of ECs’ tight junction protein disruption and encircle the Aβ-containing microvasculature [49]. Resident innate immune cells of the brain (microglia) represent about 5–12% of the brain cells [50]. Microglia are characterized by various intracellular and membranous markers, which display dissimilar levels of expression in resting and activated cells. Iba1 is microglia and macrophage-specific calcium-binding protein [51], which is substantially upregulated in reactive microglia and activated macrophages [52]. 

**Figure 6:**
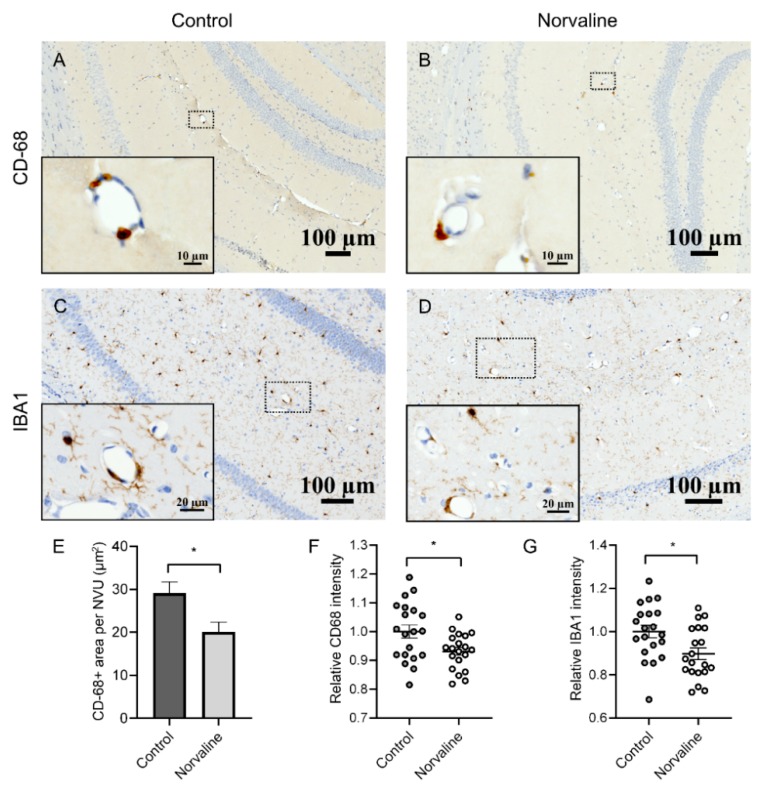
Microglia infiltrate 3×Tg-AD mice brains in a perivascular fashion. Representative hippocampal bright-field × 40 micrographs with × 100 insets. Perivascular infiltration with CD-68+ cells of control (**A**) and norvaline-treated mice (**B**) hippocampi. Iba1+ microglia in control (**C**) and norvaline-treated (**D**) mice. Measurement of the CD-68 immunopositive surface area (**E**) and stain intensity (**F**) with ZEN 2.5 revealed a significant effect of the treatment. (**G**) The relative intensity of perivascular Iba1+ microglia. Student’s unpaired t-test, *n* = 20, * *p* < 0.05.

We utilized immunohistochemistry to visualize macrophages/microglia and quantify the treatment-associated changes in the vicinity of penetrating hippocampal microvessels. We observed only a few CD-68 positive objects in each visual field of the brain parenchyma, however, there were two to three objects in the microvessels’ perivascular space (Figure 6A,B). We evidenced a significant abatement of perivascular CD-68 protein staining in the L-norvaline treated group compared to the vehicle-treated group in the 3×Tg-AD mice brains. Quantification of the area occupied by CD-68 positive objects in the vicinity of microvessels confirmed a significant reduction (by about 31%) of CD-68 immunopositive surface area (Figure 6E) and stain intensity (by about 7%) following the treatment (Figure 6F). Iba1 immunopositive area demonstrated severe variability and did not reach statistical significance; however, the level of Iba1 staining intensity was significantly diminished (by about 10%) following the treatment (Figure 6G).

### 2.3. Neuronal Nitric Oxide Synthase (nNOS) and Inducible Nitric Oxide Synthase (iNOS) mRNA Levels Are Significantly Reduced in the Brains of 3×Tg-AD Mice Compared to the WT Mice.

**Figure 7:**
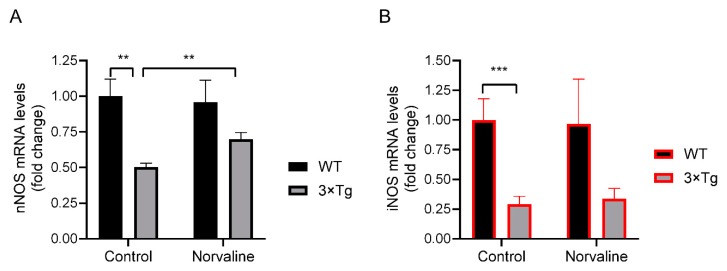
Amplification of nitric oxide synthases (NOSs) mRNA by RT-PCR. Average fold change in mRNA levels of nNOS (**A**) and iNOS (**B**) in the hippocampal tissue from WT and 3×Tg-AD mice in relation to the treatment. Bar charts represent average fold change in mRNA levels and show mean values with SEM. *n* = 6 for each group of 3×Tg-AD mice, and *n* = 3 for each group of WT mice. ** *p* < 0.01, *** *p* < 0.001.

In order to assess the brain levels of neuronal nitric oxide synthase (nNOS) and inducible nitric oxide synthase (iNOS) and disclose the strain-associated differences, we utilized real-time polymerase chain reaction (RT-PCR) methodology. The test revealed a significant (*p* < 0.01) two-fold reduction in the nNOS mRNA levels in the 3×Tg-AD mice hippocampi compared to WT mice (Figure 7A). Remarkably, the levels of iNOS showed more than three-fold significant (*p* < 0.001) reduction in the 3×Tg-AD mice compared to WT (Figure 7B). Of note, the mRNA levels of nNOS were 123 times greater than iNOS in the WT, and 234 times greater in the 3×Tg-AD mice control brains.

### 2.4. Norvaline Increases the Transcription Levels of nNOS Gene but Not iNOS Gene.

Additionally, we analyzed the nNOS and iNOS mRNA levels in the hippocampi of WT and 3×Tg-AD mice in relation to the treatment with L-norvaline. The two-way ANOVA test was used to study the effects of the genotype, the treatment, or interaction between both factors upon the levels of nNOS and iNOS. The analysis did not find any significant effect of the treatment upon the levels of iNOS in both groups of the animals (Figure 7B). Conversely, nNOS demonstrated a significant (*p* < 0.01) treatment-associated 39% growth in the 3×Tg-AD mice, without any change in the WT group (Figure 7A). Though, the interaction between the genotype and the type of treatment did not attain statistical significance.

## 3. Discussion

Converging evidence suggests a central role of endothelial NO in controlling the APP expression and processing within the brain parenchyma and vasculature. Accordingly, endothelial dysfunction caused by NO deficiency has been named a leading AD etiological factor [53]. NO is a gaseous neurotransmitter produced by nNOS, iNOS, and eNOS. The neuronal isoform (nNOS) is the primary source of NO in the CNS [54]. This enzyme is extensively expressed in the human brain, particularly in the cerebellum, hippocampus, and basal ganglia [55]. Growing evidence demonstrates co-expression of nNOS with the endothelial NOS in human ECs [56], which indicates an important role of nNOS in the ECs’ function. Accordingly, a potential anti-inflammatory role of endothelial nNOS has been hypothesized and proven [57]. Of note, nNOS deficiency leads to upregulation of iNOS and eNOS levels (two- and three-fold, respectively) in the mouse brain [58], which suggests reciprocal compensatory relationships between NOS isoforms.

Animal studies indicate an intricate role of NO in the regulation of various behaviors and the pathogenesis of psychiatric and neurodegenerative disorders [54]. Colton et al. (2008) disclosed the role of iNOS in neuroinflammation and AD [59]. They found a significant reduction of iNOS mRNA levels in human AD brains compared to age-matched healthy individuals. Accordingly, they hypothesized that AD-associated long-term exposure to Aβ leads to a decline in iNOS and consequent plunge in NO levels, below the neuroprotective threshold, which promotes Aβ-mediated neuropathology. The authors eventually proved that lack of iNOS in AD mice escalates Aβ levels, and facilitates the neurodegeneration [60].

An elegant study by Austin et al. (2010) demonstrated that NOS inhibition leads to a significant increase in the levels of APP and BACE1, which is followed by augmented Aβ secretion [61]. Moreover, the researchers evidenced significantly higher APP and BACE1 levels in the brain tissue of eNOS-deficient mice compared to WT controls. Of note, brain microvessels from these mice showed substantially higher BACE1 levels too. Consequently, the same group demonstrated that NO donor (nitroglycerin) supplementation attenuates APP and BACE1 levels in eNOS-deficient mice [62]. Furthermore, nitroglycerin significantly improved the cerebral microvessels cGMP levels in eNOS-/- mice compared to vehicle-treated mice. 

Kwak et al. (2011) found that NO and H_2_O_2_ differentially modulate BACE1 expression and enzymatic activity in vitro [63]. Importantly, the authors disclosed the mechanism and demonstrated that NO induces S-nitrosylation of BACE1, which leads to inactivation of the enzyme. Moreover, the NO/cGMP signaling had a suppressive effect upon BACE1 transcription. Dissimilarly, H_2_O_2_ induced BACE1 expression via transcriptional activation, which resulted in increased enzymatic activity [63]. Accordingly, the NO/cGMP pathway has been proposed as a promising therapeutic target [61]. 

NO has been shown to modulate the rate of leukocyte adhesion and lipid peroxidation [64]. Additionally, it regulates cerebral blood flow, modulates cells’ interaction, inhibits cysteine proteases, and enhances the antioxidative potency of reduced glutathione [65]. Though, NO displays a Janus mode of activity depending on the solvents. It terminates lipid peroxidation in an aqueous medium; however, it induces peroxidation in a non-aqueous medium [66]. In relation to AD, Chakroborty et al. (2015) have shown in an ex vivo experiment with hippocampal tissue from two-month-old 3×Tg-AD mice, that blocking NO synthesis results in a markedly augmented synaptic depression, mediated via presynaptic mechanisms [67], which points to a pivotal role of NO in synaptic function in this particular model. 

It is worth mentioning that NO possesses anti-inflammatory properties under physiological conditions. NO efficiently reduces endothelial expression of adhesion molecules and proinflammatory cytokines in vitro [68]. In contrast, reduced NO bioavailability upsurges concentrations of various inflammatory cytokines in eNOS-deficient mice brains [53]. We have shown previously that L-norvaline escalates eNOS but decreases TNFα levels in the brains of 3×Tg-AD mice [45], supposedly via NO-related mechanisms.

In the present study, we subjected to scrutiny the events happening in the vicinity of NVU and compare AD mice to WT animals, in order to decipher the mechanisms of AD development and find an adequate treatment strategy. We evaluated a promising approach for improving L-arginine and NO bioavailability in the brain tissue by inhibiting arginase with a potent inhibitor and an anti-inflammatory agent L-norvaline and investigate the BBB integrity and vascular expression of Aβ in a mouse model of AD.

**Figure 8:**
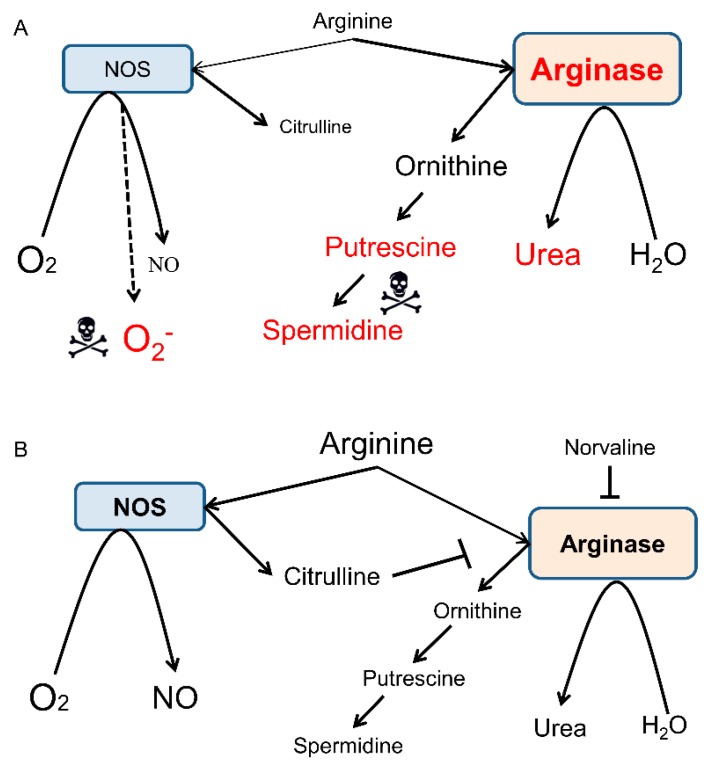
Overview of L-arginine metabolism by NOS and arginase in the AD brain. NOS oxidatively converts L-arginine into NO and citrulline, whereas arginase hydrolyzes L-arginine into ornithine and urea. These two pathways interfere with each other via complex substrate competition mechanisms. For the sake of diagram clarity, several intermediate steps, byproducts, and substrates are omitted. (**A**) Overactive arginase competes with NOS for the mutual substrate and reduces the bioavailability of L-arginine, which limits the production of NO and leads to NOS uncoupling and generation of the toxic superoxide anion. Ornithine is the precursor of putrescine and spermidine, which can be neurotoxic, in some conditions. (**B**) Application of L-norvaline inhibits arginase activity and improves L-arginine stocks [27], which provides NOS with more substrate and leads to an upsurge in NO levels. NO exerts beneficial effects upon microcirculation and APP processing. Citrulline is a byproduct of the catalyzed by NOS reaction and an arginase inhibitor. Therefore, an increase in its levels further moderates the activity of arginase.

In general, arginase inhibition improves L-arginine bioavailability [33]. L-arginine is a natural antioxidant and NOS substrate (Figure 8), which has been successfully trialed in mice [69] and demented patients [70]. However, the systemic use of L-arginine is limited due to its BBB transporter saturation under physiological conditions [71]. Moreover, L-arginine surplus upsurges the levels of polyamines, which show neurotoxicity (Figure 8A) [72,73]. 

We have demonstrated previously that L-norvaline significantly increases (by 68%) the eNOS protein levels and superoxide dismutase [Cu–Zn] (SOD1) levels in the hippocampi of 3×Tg-AD mice [45], which indicates the improvement of antioxidative brain status following arginase inhibition. Here, we adduce conclusive evidence to substantiate these results and demonstrate a more moderate (by 39%) treatment-related increase in the nNOS mRNA levels in the 3×Tg-AD mice hippocampi (Figure 7A), which further indicates improvement in L-arginine brain bioavailability following the treatment. Remarkably, we did not observe any treatment-associated alterations of nNOS levels in the brains of WT animals that point to a unique pattern of L-norvaline activity in the brain. We speculate that this inhibitor acts just on over-activated enzymes moderating its function without influencing the expression levels and activity in the WT animals. Additionally, we evidenced a significant (two-fold) decline in the hippocampal levels of nNOS mRNA in the 3×Tg-AD mice compared to WT (Figure 7B), which accords with the pattern shown by Liu et al. (2014) in human postmortem studies [74]. Of note, nNOS is coupled to the NMDA receptor complex via postsynaptic density-95 (PSD-95) protein [75]. We have shown previously that L-norvaline amplifies the expression levels of PSD-95 protein in the 3×Tg-AD mice [45], which correlates with the new findings concerning the treatment-associated increase in nNOS levels.

**Figure 9:**
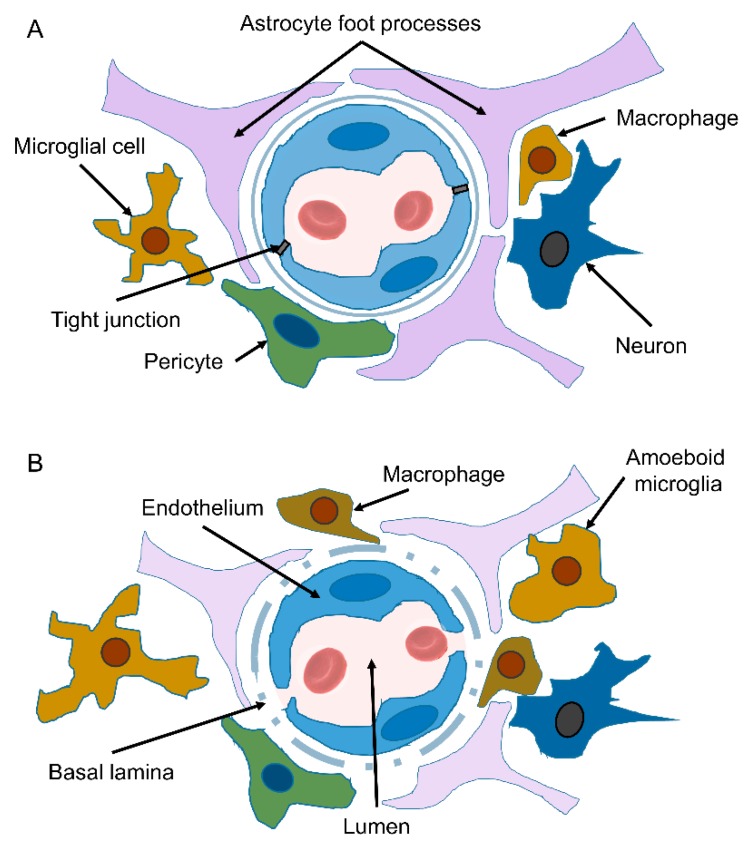
Schematic representation of the NVU at the level of brain capillary. (**A**) Endothelial cells (ECs) line the capillary lumen. They are connected via transmembrane tight junction proteins. Intercellular junctions provide tight adhesion and facilitate communication between ECs. The endothelium is separated from other cells by basal lamina (basement membrane). Astrocyte end-feet encase the vessel wall. Resting microglia with ramified phenotype survey the environment. (**B**) A model of AD-associated BBB dysfunction. Aβ deposits in the ECs that lose tight junction proteins and detach. Basal lamina disorganizes, thickens, and splits. Pericytes degenerate. Astroglia degenerate, reduce the levels of GFAP, number of extending processes, and retract their end-feet. Recruited perivascular macrophages and microglia display an activated phenotype. These alterations contribute to the diminishing of Aβ clearance, leakage of plasma proteins, and neuroinflammation.

One of the present study goals was the assessment of the CAA rate in the 3×Tg-AD mice. Several murine AD models recapitulate characteristic capillary amyloid deposition and neuroinflammation [76,77]. However, 3×Tg-AD mice have not been characterized by CAA to date. To the best of our knowledge, this is the very first study describing CAA in the 3×Tg-AD mice, which provides a comprehensive description of the pathology. Of note, CAA can appear as an independent form of angiopathy, yet it is ubiquitous in AD with about 80–90% comorbidity rate [78]. Extensive angiopathy contributes to AD-associated neurovasculature injuries, including ischemia, hemorrhages, and cortical microbleeds [12], which further aggravate the dementia symptoms (Figure 9B). Accordingly, the BBB permeability rate has been correlated with clinical dementia progression [79]. Moreover, several preclinical studies in rodent models of AD have shown the effectiveness of CAA-directed treatment upon BBB permeability rate, hemorrhage frequency, and neuroinflammation [42,80]. 

Our assay indirectly demonstrates a significant treatment-associated decrease (by 44%) in the albumin brain levels in the 3×Tg-AD mice, which points to an improvement in the BBB integrity (Figure 2). In order to decipher the mechanism of this phenomenon, we utilize a set of advanced immunohistochemistry and evidence an extensive 6E10 positivity in 3×Tg-AD mice capillary ECs (Figure 3A). Moreover, we demonstrate a significant reduction in endothelial 6E10-positive surface area and intensity as a corollary of the L-norvaline treatment (Figure 3B–D), which we relate to the increase in the levels of NOS and, subsequently, the decrease in the brain APP levels, as has been shown in our previous study [45]. 

AD clinical manifestation is followed by astrocyte morphologic alterations in humans and observed in rodent models as well [10]. Merlini et al. (2011) demonstrated an apparent retraction of astrocytic perivascular end-feet in the early presymptomatic and late-stage AD mice [81]. The authors suggest that astrocyte morphology changes and dysfunction occur at early stages of AD and contribute substantially to the development of early behavioral and cognitive deficits. In the present study, we have shown a reduction of GFAP positivity in the vicinity of the hippocampal capillary of 3×Tg-AD mice compared to WT animals, which indicates retraction of astrocytic end-feet. We evidenced a significant increase in astrocytic perivascular end-feet thickness following the treatment, which corresponds to the BBB integrity improvement in these animals (Figure 4D). Of note, 3×Tg-AD mice demonstrated a substantial reduction in astrocytic cytoskeletal branching, which indicates severe astrodegeneration [25]. We demonstrated that norvaline escalates the number of astrocytic processes in 3×Tg-AD mice (Figure 5E), which further supports the treatment-associated reversal of astrodegeneration in this model. 

Additionally, in this research, we sought to examine the role of perivascular macrophages/microglia in CAA and BBB breakdown development. Converging evidence unquestionably connects microglial responses with Aβ deposition. Moreover, microglial activation has been shown to cause wide-ranging vascular remodeling, leading to BBB insufficiency and subsequent brain parenchyma infiltration with plasma proteins [8]. Recent data demonstrated that CD68-positive microglia are consistently increased in the AD brains, though Iba1-positive cells are heterogeneous and do not show a consistent elevation [82]. Moreover, CD68-positivity rate correlates with AD-associated cognitive impairment [83]. In order to analyze the effects of L-norvaline upon macrophages/microglia, we stained the brains of 3×Tg-AD mice with Iba1 and CD-68 antibodies. In a previous study, we demonstrated a significant reduction in the hippocampal Iba1-positive microglia density, associated with a shift from activated to resting phenotype, following the treatment [27]. Here, we scrutinized Iba1 and CD-68-positive objects in the proximity of the brain microvasculature and evidenced a significant decline in the number and intensity of CD68-positive perivascular objects following the treatment (Figure 6E,F). Consequently, we suggest an essential role of the activated microglia in the BBB insufficiency.

## 4. Materials and Methods 

### 4.1. Animals and Treatment

Homozygous triple-transgenic mice (3×Tg-AD) harboring PS1(M146V), APP(Swe), and tau(P301L) transgenes were purchased from Jackson Laboratory (Bar Harbor, ME, USA) and bred in our animal facility. The 3×Tg-AD mice exhibited a synaptic dysfunction, plaque, and tangle pathology [84]. Four-month-old male 3×Tg-AD mice and age-matched male C57Bl/6 mice were divided randomly into four equal (20 mice each) groups according to their strain. The animals were housed in separate cages (five animals each) in constant ambient conditions and provided with water and food ad libitum. L-norvaline (Sigma) was dissolved in the animals’ drinking water (250 mg/L) and supplied in the animals’ cages for ten weeks in accordance with the previously published protocol [27]. The Bar-Ilan University animal care and use committee approved the experimental protocol (approval No. 82-10-2017) on October 1, 2017.

### 4.2. Evans Blue In Vivo Assay

The Evans blue dye with a molecular weight of 961 Da is a commonly used inert tracer in the BBB studies due to its strong binding capacity to serum albumin [85]. The extravasated dye can be detected by fluorescence microscopy in tissue sections and efficiently quantified by spectrophotometry or colorimetry [86]. Accordingly, the substance was shown to be a reliable marker of cerebral extravasation in rats [87] and murine models of AD [38,88]. It is a fast and straightforward method, which still represents the most commonly used test of BBB integrity [89].

In total, 200 μL Evans blue (Sigma-Aldrich; E-2129) solution in the dose of 50 µg/g were injected IP into each mouse (*n* = 6 for each group). Three hours after injection, the mice were anesthetized with ketamine (25 mg/kg) and xylazine (5 mg/kg) IP and intracardially perfused with ice-cold saline for 5 min [88]. Then, brains were dissected, weighed, and photographed intact. The brains were Dounce homogenized in one mL of 50% trichloroacetic acid and centrifuged at 13,000 rpm for 10 min. The supernatant was diluted 1:4 with 100% ethanol. The presence of the dye in the brains was measured by EPOCH2 Plate Reader/Spectrophotometer (BioTek, Winooski, Vermont, US) at an optical density of 620 nm (OD_620_).

### 4.3. Tissue Sampling

Six animals per group were rapidly decapitated with scissors. Their brains were carefully removed, and the hippocampi were dissected in accordance with the protocol published by Faraz A. Sultan [90]. The collected brain tissues were separately frozen and stored at −80 °C.

### 4.4. Fixation and Tissue Processing

Five animals from each group were deeply anesthetized with an intraperitoneal injection of Pental (0.2 mL; CTS Chemical Industries Ltd., Kiryat Malachi, Israel) and transcardially perfused with 30 mL of PBS, followed by 50 mL of chilled 4% paraformaldehyde in PBS. The brains were removed and fixed in 4% paraformaldehyde for 24 h and then transferred to 70% ethanol at 4 °C for 48 hours, dehydrated, and paraffin-embedded. The paraffin-embedded blocks were ice-cooled and sliced at a thickness of 6 µm. The sections were mounted, dried overnight at room temperature, and stored at 4 °C. 

### 4.5. Immunohistochemistry

Coronal hippocampal sections were immunolabeled to reveal the levels and location of a list of the proteins of interest. Staining was performed on a Leica Bond III system (Leica Biosystems Newcastle Ltd., Newcastle upon Tyne, UK). The tissues pretreated with an epitope-retrieval solution (Leica Biosystems Newcastle Ltd.) were incubated with primary antibodies for 30 mins in accordance with our previously published protocol [27]. A Leica Refine-HRP kit (Leica Biosystems Newcastle Ltd.) served for hematoxylin counterstaining. The omission of the primary antibodies served as a negative control.

### 4.6. Imaging and Quantification Analysis

We scrutinized the hippocampal capillaries with one endothelial cell-thick wall and 5 to 10 µm in diameter, which was predicated upon previously published data showing the mean brain capillary diameters in rodents ranging between 4.93 ± 0.29 and 5.91 ± 0.10 µm [91]. 

The parallel coronal brain sections with intervals of 25 µm were imaged using slide scanner Axio Scan.Z1 (Zeiss, Oberkochen, Germany) with a 40×/0.95 objective recording focal distances (*Z*-planes) every 0.35 μm. ZEISS Apotome.2 microscope was used to capture images with a × 100/1.4 oil immersion objective. The captured images were coded and an investigator blinded to the experimental protocol performed the examination. The morphometric cell analysis was done using ZEN Blue 2.5 software (Zeiss, Oberkochen, Germany). A fixed background intensity threshold was set for all sections representing a single type of staining. The surface of the immunoreactive area (above the selected threshold) was subjected to the statistical analysis.

The image densitometry method was used to quantify the amount of staining in the specimens. The mean grayscale optical density of individual pixels in the image, reflecting the expression levels of the proteins, was measured with digital image analysis software (Image-Pro^®^ 10.0.1; Media Cybernetics, Inc., Rockville, MD, USA), validated with ImageJ (NIH), and presented as the average value ±SEM for each treatment group. Imaris 9.3 (Oxford Instruments plc, Abingdon, UK) was used to create a 3D image reconstruction and quantify the cell body volume, stain intensity, and the number of astroglia processes. 

#### 4.6.1. 6E10 Staining and Analysis

For the quantitative histochemical analysis of β-amyloid, two coronal brain sections per mouse cut at 25 µm intervals throughout the hippocampi (1.6–1.7 mm posterior to bregma) were used. Immunohistochemistry was performed on the plane-matched coronal sections. Primary 6E10 (Abcam, #ab2539) antibodies with dilution 1:200 were utilized, as published previously [45]. We analyzed the 6E10+ surface area and optical density of the hippocampal penetrating blood microvessels (about 10 µm in diameter) with ZEN 2.5. The immunopositive surface area above the preset threshold was divided by the area of the biggest representing the vessel circle in µm^2^. Then, relative numbers, which reflect the adjusted immunopositivity, were subjected to statistical analysis.

#### 4.6.2. GFAP Staining and Analysis

We analyzed the astrocytic cytoskeletal genotype and treatment-related changes within the hippocampal NVU of 3×Tg-AD and WT mice by measuring the surface area, intensity, and processes number of GFAP positive profiles. For the quantitative histochemical analysis, two coronal brain sections per mouse, cut at 25 µm intervals throughout the hippocampi (1.9–2.0 mm posterior to bregma), were used. The anti-GFAP antibody (Biolegend, #835301) with dilution 1:1000 served to determine the glial cytoskeletal profiles and treatment-related changes. Parallel confocal planes with step of 0.35 µm were scrutinized to characterize morphological features of the GFAP-positive objects. The NVU-associated astroglial end-feet were evaluated with ZEN 2.5. The end-feet thickness was measured (four points per one NVU), averaged, and tested statistically. Two representative (clearly seen in each plane) cells (each animal) from Cornus Ammonis 1 (CA1) hippocampal area were chosen for 3D reconstruction and analysis with Imaris 9.3 (Oxford Instruments plc, Abingdon, UK). The cell body volume, stain intensity, and number of astroglia processes were quantified and compared statistically.

#### 4.6.3. Iba1 and CD-68 Staining and Analysis

We studied the microglial ionized calcium-binding adaptor molecule 1 (Iba1) immunoreactivity in the vicinity of small hippocampal blood vessels. Perivascular microglia were subjected to the analysis of their density, immunopositive surface area, and intensity. The Iba1 antibody (Novus Biologicals, #NB100-1028) with dilution 1:500 was used as a marker of macrophage/microglia. In order to study the rate of microglial activation, the levels of the microglial marker CD-68 were quantified. Rabbit polyclonal CD-68 antibody (Abcam, #ab125212) with dilution 1:200 was utilized. Of note, CD-68 is a lysosomal protein that is commonly used as a marker of reactive microglia [92].

### 4.7. RNA Isolation and Reverse Transcription

Total RNA was isolated from the hippocampi using the RNeasy Mini Kit (Cat# 74104, QIAGEN, Hilden, Germany) following the manufacturer’s instructions, including DNAase treatment. RNA quantification was performed using QubitTM RNA HS Assay Kit (Cat# Q32852, Invitrogen, Carlsbad, CA, USA). RNA integrity (RIN) was measured using Agilent 2100 Bioanalyzer System and Agilent RNA 6000 Pico Kit (Cat# 5067-1513, Agilent Technologies, Santa Clara, CA, USA). cDNA was prepared from 200 ng of total RNA using SuperScript® III First-Strand Synthesis System for real-time polymerase chain reaction (RT-PCR) (Cat#18080-051, Invitrogen, Carlsbad, CA, USA) following the manufacturer’s instructions.

### 4.8. Polymerase Chain Reaction Amplification

The mRNA expression levels of neuronal and inducible nitric oxide synthases (nNOS, iNOS) were detected as described in our previous study [45]. Original TaqMan™ (Thermo Fisher Scientific, Waltham, MA, USA) primers Mm01208059_m1 and Mm00440502_m1 were utilized. ACTB probe (Mm00607939_s1) served as an endogenous housekeeping gene for normalization of RNA levels. PCR was set in triplicates following the manufacturer’s instructions (Applied Biosystems, Insert PN 4444602 Rev. C) in 10 μL volume using five ng of cDNA template. PCR data were analyzed in the StepOnePlus system installed with StepOne Software v2.3 (Applied Biosystems). The quantification was performed using the comparative *C*_t_ (ΔΔ*C*_t_) method. 

### 4.9. Statistical Analysis

Statistical analyses were conducted with GraphPad Prism 8.0 for Windows (GraphPad Software, San Diego, CA, USA). The significance was set at 95% of confidence. The two-way ANOVA test was used to demonstrate whether the genotype, the treatment, or the interaction between both factors had an impact upon the phenotype. The two-tailed Student’s *t*-test was performed to compare the means of two groups. The Kolmogorov–Smirnov test served to evaluate the normality of the data distribution. All data are presented as mean values. Throughout the text and in plots, the variability is indicated by the standard error of the mean (SEM).

## 5. Conclusions

In the present study, we have sifted the evidence of L-norvaline neuroprotective properties with emphasis upon the BBB integrity in a murine model of AD. The overall results indicate the multifarious effects of L-norvaline upon NVU function in 3×Tg-AD mice. Of note, this substance shows no significant effects in WT animals. Remarkably, in another recent study, L-norvaline did not influence blood pressure in WT rats; however, it significantly reduced this parameter in hypertensive animals [93]. For that reason, we argue that L-norvaline tunes arginase activity in regions with its overactivation and, in only the compartments with L-arginine deficiency, improves its bioavailability. However, ancillary evidence is needed to support our hypothesis. 

## Abbreviations

AD	Alzheimer’s disease
BBB	blood-brain barrier
3×Tg-AD	triple-transgenic mouse model of Alzheimer’s disease
NOS	nitric oxide synthase
Aβ	amyloid-beta
NFT	neurofibrillary tangles
CAA	cerebral amyloid angiopathy
APP	amyloid precursor protein
CNS	central nervous system
CSF	cerebrospinal fluid
ECs	endothelial cells
CNS	central nervous system
CSF	cerebrospinal fluid
NVU	neurovascular unit
BACE1	β-site APP cleaving enzyme 1
WT	wild-type
OD	optical density
ANOVA	analysis of variance
CA	cornus Ammonis
SEM	standard error of the mean
CD-68	cluster of differentiation 68
Iba1	ionized calcium-binding adapter molecule 1
mRNA	messenger ribonucleic acid
PSD-95	postsynaptic density protein 95
GFAP	glial fibrillary acidic protein

## References

[1] Alzheimer’s Association (2016). 2016 Alzheimer’s Disease Facts and Figures. Alzheimer’s Dement..

[2] Goedert M., Spillantini M.G. (2006). A century of Alzheimer’s disease. Science.

[3] Thal D.R., Griffin W.S.T., de Vos R.A.I., Ghebremedhin E. (2008). Cerebral amyloid angiopathy and its relationship to Alzheimer’s disease. Acta Neuropathol..

[4] Kim J., Jeong Y. (2015). In vivo Image of Cerebral Amyloid Angiopathy in an Alzheimer’s Disease Mouse Model. J. Stroke.

[5] Van Dorpe J.A., Smeijers L., Dewachter I., Kurt S., Van Den Haute C., Tesseur I., Sciot R., Van Leuven F. (2003). Prominent cerebral amyloid angiopathy in transgenic mice overexpressing the London mutant of human APP in neurons. Neurobiol. Aging.

[6] Daneman R. (2012). The blood-brain barrier in health and disease. Ann. Neurol..

[7] Roher A.E., Tyas S.L., Maarouf C.L., Daugs I.D., Kokjohn T.A., Emmerling M.R., Garami Z., Belohlavek M., Sabbagh M.N., Sue L.I. (2011). Intracranial atherosclerosis as a contributing factor to Alzheimer’s disease dementia. Alzheimer’s Dement..

[8] Ryu J.K., McLarnon J.G. (2009). A leaky blood-brain barrier, fibrinogen infiltration and microglial reactivity in inflamed Alzheimer’s disease brain. J. Cell. Mol. Med..

[9] Erickson M.A., Banks W.A. (2013). Blood-brain barrier dysfunction as a cause and consequence of Alzheimer’s disease. J. Cereb. Blood Flow Metab..

[10] Yamazaki Y., Kanekiyo T. (2017). Blood-brain barrier dysfunction and the pathogenesis of alzheimer’s disease. Int. J. Mol. Sci..

[11] Arvanitakis Z., Leurgans S.E., Wang Z., Wilson R.S., Bennett D.A., Schneider J.A. (2011). Cerebral amyloid angiopathy pathology and cognitive domains in older persons. Ann. Neurol..

[12] Hartz A.M.S., Bauer B., Soldner E.L.B., Wolf A., Boy S., Backhaus R., Mihaljevic I., Bogdahn U., Klünemann H.H., Schuierer G. (2012). Amyloid-β contributes to blood-brain barrier leakage in transgenic human amyloid precursor protein mice and in humans with cerebral amyloid angiopathy. Stroke.

[13] Greenberg S.M., Gurol M.E., Rosand J., Smith E.E. (2004). Amyloid angiopathy-related vascular cognitive impairment. Stroke.

[14] Pfeifer L.A., White L.R., Ross G.W., Petrovitch H., Launer L.J. (2002). Cerebral amyloid angiopathy and cognitive function: The HAAS autopsy study. Neurology.

[15] Daneman R., Prat A. (2015). The Blood–Brain Barrier. Cold Spring Harb. Perspect. Biol..

[16] Abbott N.J., Patabendige A.A.K., Dolman D.E.M., Yusof S.R., Begley D.J. (2010). Structure and function of the blood-brain barrier. Neurobiol. Dis..

[17] Attwell D., Buchan A.M., Charpak S., Lauritzen M., Macvicar B.A., Newman E.A. (2010). Glial and neuronal control of brain blood flow. Nature.

[18] Muoio V., Persson P.B., Sendeski M.M. (2014). The neurovascular unit—Concept review. Acta Physiol..

[19] Liu C.Y., Yang Y., Ju W.N., Wang X., Zhang H.L. (2018). Emerging Roles of Astrocytes in Neuro-Vascular Unit and the Tripartite Synapse With Emphasis on Reactive Gliosis in the Context of Alzheimer’s Disease. Front. Cell. Neurosci..

[20] Frost G.R., Li Y.M. (2017). The role of astrocytes in amyloid production and Alzheimer’s disease. Open Biol..

[21] Liu C.C., Hu J., Zhao N., Wang J., Wang N., Cirrito J.R., Kanekiyo T., Holtzman D.M., Bu G. (2017). Astrocytic LRP1 Mediates Brain Aβ Clearance and Impacts Amyloid Deposition. J. Neurosci..

[22] Verkhratsky A., Olabarria M., Noristani H.N., Yeh C.Y., Rodriguez J.J. (2010). Astrocytes in Alzheimer’s Disease. Neurotherapeutics.

[23] Heneka M.T., Sastre M., Dumitrescu-Ozimek L., Dewachter I., Walter J., Klockgether T., Van Leuven F. (2005). Focal glial activation coincides with increased BACE1 activation and precedes amyloid plaque deposition in APP[V717I] transgenic mice. J. Neuroinflamm..

[24] Price B.R., Norris C.M., Sompol P., Wilcock D.M. (2018). An emerging role of astrocytes in vascular contributions to cognitive impairment and dementia. J. Neurochem..

[25] Kulijewicz-Nawrot M., Verkhratsky A., Chvátal A., Syková E., Rodríguez J.J. (2012). Astrocytic cytoskeletal atrophy in the medial prefrontal cortex of a triple transgenic mouse model of Alzheimer’s disease. J. Anat..

[26] Olabarria M., Noristani H.N., Verkhratsky A., Rodríguez J.J. (2010). Concomitant astroglial atrophy and astrogliosis in a triple transgenic animal model of Alzheimer’s disease. Glia.

[27] Polis B., Srikanth K.D., Elliott E., Gil-Henn H., Samson A.O. (2018). L-Norvaline Reverses Cognitive Decline and Synaptic Loss in a Murine Model of Alzheimer’s Disease. Neurotherapeutics.

[28] Stollg G., Jander S. (1999). The role of microglia and macrophages in the pathophysiology of the CNS. Prog. Neurobiol..

[29] Zaghi J., Goldenson B., Inayathullah M., Lossinsky A.S., Masoumi A., Avagyan H., Mahanian M., Bernas M., Weinand M., Rosenthal M.J. (2009). Alzheimer disease macrophages shuttle amyloid-beta from neurons to vessels, contributing to amyloid angiopathy. Acta Neuropathol..

[30] Hawkes C.A., McLaurin J. (2009). Selective targeting of perivascular macrophages for clearance of amyloid in cerebral amyloid angiopathy. Proc. Natl. Acad. Sci. USA.

[31] Park L., Uekawa K., Garcia-Bonilla L., Koizumi K., Murphy M., Pistik R., Younkin L., Younkin S., Zhou P., Carlson G. (2017). Brain Perivascular Macrophages Initiate the Neurovascular Dysfunction of Alzheimer Aβ Peptides. Circ. Res..

[32] Kisumi M., Sugiura M., Takagi T., Chibata I. (1977). Norvaline accumulation by regulatory mutants of Serratia marcescens. J. Antibiot..

[33] Polis B., Samson A.O. (2018). Arginase as a Potential Target in the Treatment of Alzheimer’s Disease. Adv. Alzheimer’s Dis..

[34] Ming X.F., Rajapakse A.G., Carvas J.M., Ruffieux J., Yang Z. (2009). Inhibition of S6K1 accounts partially for the anti-inflammatory effects of the arginase inhibitor L-norvaline. BMC Cardiovasc. Disord..

[35] Pernow J., Jung C. (2013). Arginase as a potential target in the treatment of cardiovascular disease: Reversal of arginine steal?. Cardiovasc. Res..

[36] Montagne A., Barnes S.R., Sweeney M.D., Halliday M.R., Sagare A.P., Zhao Z., Toga A.W., Jacobs R.E., Liu C.Y., Amezcua L. (2015). Blood-Brain barrier breakdown in the aging human hippocampus. Neuron.

[37] van de Haar H.J., Jansen J.F.A., van Osch M.J.P., van Buchem M.A., Muller M., Wong S.M., Hofman P.A.M., Burgmans S., Verhey F.R.J., Backes W.H. (2016). Neurovascular unit impairment in early Alzheimer’s disease measured with magnetic resonance imaging. Neurobiol. Aging.

[38] Montagne A., Zhao Z., Zlokovic B.V. (2017). Alzheimer’ s disease: A matter of blood—Brain barrier dysfunction?. J. Exp. Med..

[39] Tibbling G., Link H., Öhman S. (1977). Principles of albumin and igg analyses in neurological disorders. I. Establishment of reference values. Scand. J. Clin. Lab. Investig..

[40] Elovaara I., Icen A., Palo J., Erkinjuntti T. (1985). CSF in Alzheimer’s disease. Studies on blood-brain barrier function and intrathecal protein synthesis. J. Neurol. Sci..

[41] Alafuzoff I., Adolfsson R., Bucht G., Winblad B. (1983). Albumin and immunoglobulin in plasma and cerebrospinal fluid, and blood-cerebrospinal fluid barrier function in patients with dementia of alzheimer type and multi-infarct dementia. J. Neurol. Sci..

[42] Ezra A., Rabinovich-Nikitin I., Rabinovich-Toidman P., Solomon B. (2015). Multifunctional Effect of Human Serum Albumin Reduces Alzheimer’s Disease Related Pathologies in the 3xTg Mouse Model. J. Alzheimer’s Dis..

[43] d’Uscio L.V., He T., Katusic Z.S. (2017). Expression and Processing of Amyloid Precursor Protein in Vascular Endothelium. Physiology.

[44] Kitazume S., Tachida Y., Kato M., Yamaguchi Y., Honda T., Hashimoto Y., Wada Y., Saito T., Iwata N., Saido T. (2010). Brain endothelial cells produce amyloid β from amyloid precursor protein 770 and preferentially secrete the O-glycosylated form. J. Biol. Chem..

[45] Polis B., Srikanth K., Gurevich V., Gil-Henn H., Samson A. (2019). L-Norvaline, a new therapeutic agent against Alzheimer’s disease. Neural Regen. Res..

[46] Prat A., Biernacki K., Wosik K., Antel J.P. (2001). Glial cell influence on the human blood-brain barrier. Glia.

[47] Nedergaard M., Ransom B., Goldman S.A. (2003). New roles for astrocytes: Redefining the functional architecture of the brain. Trends Neurosci..

[48] Verkhratsky A., Parpura V. (2016). Astrogliopathology in neurological, neurodevelopmental and psychiatric disorders. Neurobiol. Dis..

[49] Fiala M., Liu Q.N., Sayre J., Pop V., Brahmandam V., Graves M.C., Vinters H.V. (2002). Cyclooxygenase-2-positive macrophages infiltrate the Alzheimer’s disease brain and damage the blood-brain barrier. Eur. J. Clin. Investig..

[50] Mittelbronn M., Dietz K., Schluesener H.J., Meyermann R. (2001). Local distribution of microglia in the normal adult human central nervous system differs by up to one order of magnitude. Acta Neuropathol..

[51] Ito D., Imai Y., Ohsawa K., Nakajima K., Fukuuchi Y., Kohsaka S. (1998). Microglia-specific localisation of a novel calcium binding protein, Iba1. Mol. Brain Res..

[52] Lan X., Han X., Li Q., Yang Q.W., Wang J. (2017). Modulators of microglial activation and polarization after intracerebral haemorrhage. Nat. Rev. Neurol..

[53] Austin S.A., Santhanam A.V., Hinton D.J., Choi D.S., Katusic Z.S. (2013). Endothelial nitric oxide deficiency promotes Alzheimer’s disease pathology. J. Neurochem..

[54] Freudenberg F., Alttoa A., Reif A. (2015). Neuronal nitric oxide synthase (NOS1) and its adaptor, NOS1AP, as a genetic risk factors for psychiatric disorders. Genes Brain Behav..

[55] Blum-Degen D., Heinemann T., Lan J., Pedersen V., Leblhuber F., Paulus W., Riederer P., Gerlach M. (1999). Characterization and regional distribution of nitric oxide synthase in the human brain during normal ageing. Brain Res..

[56] Bachetti T., Comini L., Curello S., Bastianon D., Palmieri M., Bresciani G., Callea F., Ferrari R. (2004). Co-expression and modulation of neuronal and endothelial nitric oxide synthase in human endothelial cells. J. Mol. Cell. Cardiol..

[57] Chakrabarti S., Chan C.K., Jiang Y., Davidge S.T. (2012). Neuronal nitric oxide synthase regulates endothelial inflammation. J. Leukoc. Biol..

[58] Finkel J., Guptill V., Khaibullina A., Spornick N., Vasconcelos O., Liewehr D.J., Steinberg S.M., Quezado Z.M.N. (2012). The three isoforms of nitric oxide synthase distinctively affect mouse nocifensive behavior. Nitric Oxide Biol. Chem..

[59] Colton C.A., Wilcock D.M., Wink D.A., Davis J., Van Nostrand W.E., Vitek M.P. (2008). The effects of NOS2 gene deletion on mice expressing mutated human AβPP. J. Alzheimer’s Dis..

[60] Colton C.A., Vitek M.P., Wink D.A., Xu Q., Cantillana V., Previti M.L., Van Nostrand W.E., Weinberg J.B., Dawson H. (2006). NO synthase 2 (NOS2) deletion promotes multiple pathologies in a mouse model of Alzheimer’s disease. Proc. Natl. Acad. Sci. USA.

[61] Austin S.A., Santhanam A.V., Katusic Z.S. (2010). Endothelial nitric oxide modulates expression and processing of amyloid precursor protein. Circ. Res..

[62] Austin S.A., D’uscio L.V., Katusic Z.S. (2013). Supplementation of Nitric Oxide Attenuates AβPP and BACE1 Protein in Cerebral Microcirculation of eNOS-Deficient Mice. J. Alzheimer’s Dis..

[63] Kwak Y.D., Wang R., Li J.J., Zhang Y.W., Xu H., Liao F.F. (2011). Differential regulation of BACE1 expression by oxidative and nitrosative signals. Mol. Neurodegener..

[64] Wink D.A., Miranda K.M., Espey M.G., Pluta R.M., Hewett S.J., Colton C., Vitek M., Feelisch M., Grisham M.B. (2002). Mechanisms of the Antioxidant Effects of Nitric Oxide. Antioxid. Redox Signal..

[65] Chiueh C.C. (1999). Neuroprotective properties of nitric oxide. Ann. N. Y. Acad. Sci..

[66] Hiramoto K., Ohkawa T., Oikawa N., Kikugawa K. (2003). Is Nitric Oxide (NO) an Antioxidant or a Prooxidant for Lipid Peroxidation?. Chem. Pharm. Bull..

[67] Chakroborty S., Kim J., Schneider C., West A.R., Stutzmann G.E. (2015). Nitric Oxide Signaling Is Recruited As a Compensatory Mechanism for Sustaining Synaptic Plasticity in Alzheimer’s Disease Mice. J. Neurosci..

[68] De Caterina R., Libby P., Peng H.B., Thannickal V.J., Rajavashisth T.B., Gimbrone M.A., Shin W.S., Liao J.K. (1995). Nitric oxide decreases cytokine-induced endothelial activation: Nitric oxide selectively reduces endothelial expression of adhesion molecules and proinflammatory cytokines. J. Clin. Investig..

[69] Fonar G., Polis B., Meirson T., Maltsev A., Elliott E., Samson A.O. (2018). Intracerebroventricular administration of L-arginine improves spatial memory acquisition in triple transgenic mice via reduction of oxidative stress and apoptosis. Transl. Neurosci..

[70] Ohtsuka Y., Nakaya J. (2000). Effect of oral administration of L-arginine on senile dementia. Am. J. Med..

[71] Tachikawa M., Hosoya K. (2011). Transport characteristics of guanidino compounds at the blood-brain barrier and blood-cerebrospinal fluid barrier: relevance to neural disorders. Fluids Barriers CNS.

[72] de Vera N., Serratosa J., Artigas F., Martínez E. (1992). Toxic effects of putrescine in rat brain: Polyamines can be involved in the action of excitotoxins. Amino Acids.

[73] Sparapani M., Dall’Olio R., Gandolfi O., Ciani E., Contestabile A. (1997). Neurotoxicity of Polyamines and Pharmacological Neuroprotection in Cultures of Rat Cerebellar Granule Cells. Exp. Neurol..

[74] Liu P., Fleete M.S., Jing Y., Collie N.D., Curtis M.A., Waldvogel H.J., Faull R.L.M., Abraham W.C., Zhang H. (2014). Altered arginine metabolism in Alzheimer’s disease brains. Neurobiol. Aging.

[75] Nedvetsky P.I., Sessa W.C., Schmidt H.H.H.W. (2002). There’s NO binding like NOS binding: Protein-protein interactions in NO/cGMP signaling. Proc. Natl. Acad. Sci. USA.

[76] Fryer J.D. (2005). Human Apolipoprotein E4 Alters the Amyloid 40:42 Ratio and Promotes the Formation of Cerebral Amyloid Angiopathy in an Amyloid Precursor Protein Transgenic Model. J. Neurosci..

[77] Van Nostrand W.E., Xu F., Rozemuller A.J.M., Colton C.A. (2010). Enhanced capillary amyloid angiopathy-associated pathology in Tg-SwDI mice with deleted nitric oxide synthase 2. Stroke.

[78] Serrano-Pozo A., Frosch M.P., Masliah E., Hyman B.T. (2011). Neuropathological alterations in Alzheimer disease. Cold Spring Harb. Perspect. Med..

[79] Wada H. (2008). Blood-Brain Barrier Permeability of the Demented Elderly as Studied by Cerebrospinal Fluid-Serum Albumin Ratio. Intern. Med..

[80] Yan P., Zhu A., Liao F., Xiao Q., Kraft A.W., Gonzales E., Perez R., Greenberg S.M., Holtzman D.M., Lee J.M. (2015). Minocycline Reduces Spontaneous Hemorrhage in Mouse Models of Cerebral Amyloid Angiopathy. Stroke.

[81] Merlini M., Meyer E.P., Ulmann-Schuler A., Nitsch R.M. (2011). Vascular β-amyloid and early astrocyte alterations impair cerebrovascular function and cerebral metabolism in transgenic arcAβ mice. Acta Neuropathol..

[82] Hopperton K.E., Mohammad D., Trépanier M.O., Giuliano V., Bazinet R.P. (2018). Markers of microglia in post-mortem brain samples from patients with Alzheimer’s disease: A systematic review. Mol. Psychiatry.

[83] Minett T., Classey J., Matthews F.E., Fahrenhold M., Taga M., Brayne C., Ince P.G., Nicoll J.A.R., Boche D. (2016). Microglial immunophenotype in dementia with Alzheimer’s pathology. J. Neuroinflamm..

[84] Oddo S., Caccamo A., Shepherd J.D., Murphy M.P., Golde T.E., Kayed R., Metherate R., Mattson M.P., Akbari Y., LaFerla F.M. (2003). Triple-transgenic model of Alzheimer’s Disease with plaques and tangles: Intracellular Aβ and synaptic dysfunction. Neuron.

[85] Wolman M., Klatzo I., Chui E., Wilmes F., Nishimoto K., Fujiwara K., Spatz M. (1981). Evaluation of the dye-protein tracers in pathophysiology of the blood-brain barrier. Acta Neuropathol..

[86] Uyama O., Okamura N., Yanase M., Narita M., Kawabata K., Sugita M. (1988). Quantitative evaluation of vascular permeability in the gerbil brain after transient ischemia using Evans blue fluorescence. J. Cereb. Blood Flow Metab..

[87] Yen L.F., Wei V.C., Kuo E.Y., Lai T.W. (2013). Distinct Patterns of Cerebral Extravasation by Evans Blue and Sodium Fluorescein in Rats. PLoS ONE.

[88] Ujiie M., Dickstein D.L., Carlow D.A., Jefferies W.A. (2003). Blood-brain barrier permeability precedes senile plaque formation in an Alzheimer disease model. Microcirculation.

[89] Yao L., Xue X., Yu P., Ni Y., Chen F. (2018). Evans Blue Dye: A Revisit of Its Applications in Biomedicine. Contrast Media Mol. Imaging.

[90] Sultan F. (2016). Dissection of Different Areas from Mouse Hippocampus. BIO-PROTOCOL.

[91] Duelli R., Kuschinsky W. (1993). Changes in brain capillary diameter during hypocapnia and hypercapnia. J. Cereb. Blood Flow Metab..

[92] Zhao X., Liao Y., Morgan S., Mathur R., Feustel P., Mazurkiewicz J., Qian J., Chang J., Mathern G.W., Adamo M.A. (2018). Noninflammatory Changes of Microglia Are Sufficient to Cause Epilepsy. Cell Rep..

[93] Gilinsky M.A., Polityko Y.K., Markel A.L., Latysheva T.V., Samson A.O., Polis B., Naumenko S.E. (2019). Norvaline reduces blood pressure and induces diuresis in rats with inherited stress-induced arterial hypertension. bioRxiv.

